# Systematic review of acupuncture to improve ovarian function in women with poor ovarian response

**DOI:** 10.3389/fendo.2023.1028853

**Published:** 2023-03-13

**Authors:** Rong-Rong Wang, Meng-Hua Su, Li-Ying Liu, Yuan-Yuan Lai, Xiao-Li Guo, Di Gan, Xiao-Yan Zheng, Han Yang, Si-Yi Yu, Fan-Rong Liang, Wei Wei, Ying Zhong, Jie Yang

**Affiliations:** ^1^ Acupuncture and Tuina School, Chengdu University of Traditional Chinese Medicine, Chengdu, Sichuan, China; ^2^ Traditional Chinese Medicine Department, Chengdu Xi'nan Gynecology Hospital, Chengdu, Sichuan, China; ^3^ Clinical Research Center for Acupuncture and Moxibustion in Sichuan Province, Chengdu, Sichuan, China

**Keywords:** acupuncture, poor ovarian response, ovarian function, *in vitro* fertilization, systematic review

## Abstract

**Objective:**

To determine the effect of acupuncture in treating poor ovarian response (POR).

**Methods:**

We searched MEDLINE (via PubMed), EMBASE, Allied and Complementary Medicine Database, CNKI, CBM, VIP database, Wanfang Database, and relevant registration databases from inception to January 30, 2023. In this review, both Chinese and English peer-reviewed literature were included. Only randomized controlled trials (RCTs) using acupuncture as an intervention for POR patients undergoing *in vitro* fertilization were considered.

**Results:**

Seven clinical randomized controlled trials (RCTs) were eventually included for comparison (516 women). The quality of included studies was generally low or very low. For the meta-analysis, seven studies showed that compared with controlled ovarian hyperstimulation (COH) therapy, acupuncture combined with COH therapy could significantly increase the implantation rate (RR=2.13, 95%CI [1.08, 4.21], *p*=0.03), the number of oocytes retrieved (MD=1.02, 95%CI [0.72, 1.32], *p*<0.00001), the thickness of endometrium (MD=0.54, 95%CI [0.13, 0.96], *p*=0.01), and the antral follicle count (MD=1.52, 95%CI [1.08, 1.95], *p*<0.00001), reduce follicle-stimulating hormone (FSH) levels (MD=-1.52, 95%CI [-2.41, -0.62], *p*=0.0009) and improve estradiol (E_2_) levels (MD=1667.80, 95%CI [1578.29, 1757.31], *p*<0.00001). Besides, there were significant differences in the duration of Gn (MD=0.47, 95%CI [-0.00, 0.94], *p*=0.05) between the two groups. However, no statistical variation was observed in improving clinical pregnancy rate (CPR), fertilization rate, high-quality embryo rate, luteinizing hormone (LH) value, anti-mullerian hormone (AMH) value, or reducing the dose of gonadotropin (Gn) values between the acupuncture plus COH therapy group and the COH therapy group.

**Conclusion:**

Acupuncture combined with COH therapy is doubtful in improving the pregnancy outcome of POR patients. Secondly, acupuncture can also improve the sex hormone level of POR women, and improve ovarian function. Furthermore, more RCTs of acupuncture in POR are needed to be incorporated into future meta-analyses.

**Systematic review registration:**

PROSPERO, identifier CRD42020169560.

## Introduction

Poor ovarian response (POR) is a pathological condition characterized by a low ovarian response to controlled ovarian stimulation (1, 2). Among women who receive controlled ovarian hyperstimulation (COH), the incidence of POR has reached 9%-24% (3). Several studies indicated that only 33%-40% of women receiving *in vitro* fertilization-embryo transfer (IVF-ET) achieve a clinical pregnancy (4), whereas the clinical pregnancy rate (CPR) for POR patients is approximately 2%-18% (5). The high occurrence of POR hampers the success of assisted reproductive technology.

POR is mainly manifested as fewer follicles during COH, a lower blood estrogen peak, a higher dose of gonadotropin (Gn), a higher cycle cancellation rate, fewer oocytes, and a lower CPR (6). Growing evidence demonstrated that POR is directly associated with women’s age (1, 7, 8). Advanced age is one of the most important causes of POR (9). With age, the number of follicles in the ovary gradually decreased, the quality of oocytes decreased, and the ovarian response to Gn decreased (2, 10, 11), affecting female pregnancy outcomes. For patients with POR risk factors and a POR diagnosis, the key to the success of first-cycle and multi-cycle stimulation are the accurate evaluation of ovarian reserve function and the adoption of a tailored ovarian stimulation protocol (12). The gonadotropin-releasing hormone (GnRH) long protocol, GnRH short protocol, or GnRH antagonist protocol (13) were selected based on how well the patient’s ovarian reserve functioned and how long it had been since their last ovulation cycle. IVF-ET has completely changed how infertile couples are treated, but its success rate is not as good as it could be. Due to the complexity of the clinical situation and the high cost of treatment, some couples are also looking for subsidies or alternative therapies to improve the effectiveness of IVF-ET treatment.

Acupuncture has been widely used in IVF-ET and has made some progress in improving the clinical outcomes in terms of pregnancy (14–17). Clinical practice has shown that acupuncture can benignly regulate the secretion function of the hypothalamus-pituitary-ovarian axis and hypothalamus-pituitary-adrenal axis in patients with POR (18–20), to promote the secretion of Gn tend to be normal, as well as to improve the ovarian reserve function and oocyte quality. Several studies (21) have also confirmed that transcutaneous electrical acupoint stimulation (TEAS) can improve ovarian blood supply and ovarian responsibility in patients with POR. However, whether acupuncture-assisted treatment of POR can improve the CPR of IVF-ET patients has always been controversial (22, 23). Although there has been a review (22) on the effectiveness of acupuncture in patients with POR, the effectiveness of acupuncture is doubted due to the small number of studies, poor quality, and few outcome indicators. Therefore, acupuncture used as an alternative therapy in POR patients during IVF-ET needs to be evaluated comprehensively and systematically.

This review provided clinicians with more precise data on the effectiveness of acupuncture for POR patients during COH in the context of IVF as well as identified areas where further evaluation is necessary.

## Method

The review was registered at PROSPERO. All contents and report details were strictly referred to as Preferred Reporting Items for Systematic Reviews and Meta-analyses (PRISMA) (24).

### Search strategy

Several databases have been searched such as MEDLINE (via PubMed), EMBASE, Allied and Complementary Medicine Database, CNKI, CBM, VIP database, and Wanfang Database from their inception to January 30, 2023. And we also searched four clinical trial registries: Menstrual Disorders and Subfertility Group Specialized Register, Cochrane Central Register of Controlled Trials, World Health Organization International Clinical Trials Registry Platform, Chinese Clinical Registry, and Clinical Trials. Both Chinese and English literature were embedded. The search strategy was constructed by subject words and free words, such as “poor ovarian response”, “poor ovarian responder”, and “controlled ovarian hyperstimulation”. The search words in the databases were adjusted according to the specific database. The search strategies were shown in **Supplementary Material.**

### Inclusion criteria

We included RCTs evaluating the efficacy of acupuncture for POR. The diagnostic criteria of POR were not limited but identified. Subjects were women with POR receiving IVF. There were no restrictions on the age or race of enrolled subjects. All POR patients were required to receive acupuncture during IVF.

Acupuncture therapy included hand acupuncture, auricular acupuncture, TEAS, electroacupuncture, acupoint embedding, and acupoint injection. We excluded studies comparing the effects on different types of acupoints or forms of acupuncture. There was no limit to the duration of intervention for treatment, but it should be recorded in detail.

The following outcomes for acupuncture treating POR were included.

1) Primary outcome
CPR.
2) Secondary outcome
Other indicators of pregnancy outcome: Fertilization rate, number of oocytes retrieved per woman, high-quality embryo rate, implantation rate, thickness of endometrium, and cycle cancellation rate
Index for reproductive endocrinology: the value of follicle-stimulating hormone (FSH), luteinizing hormone (LH), estradiol (E_2_), antral follicle count (AFC), anti-mullerian hormone (AMH), dose and duration of Gn used for COH.
3) Adverse effect
Miscarriage rate.

### Data selection and collection

Two independent authors (LYL and WW) screened the titles and abstracts of the searched studies after excluding duplication. Excluded studies listed the reasons for exclusion in the table of Microsoft Excel 2016. Any disagreement between two authors was arbitrated by another author (RRW). The following items of included trials were extracted by two authors independently into a predefined data extraction sheet: general information (article ID author list, year of publication, title, journal name), the methodology of included trials (participant demographics, eligibility criteria, interventions, controls, follow-up measurement), outcomes measurement.

### Study risk of bias assessment

We generated risk of bias maps using Review Manager (RevMan, version 5.3). The Cochrane risk of bias assessment tool (25) was used to assess the six risk of bias entries in the included literature. The content included the generation of random sequences generation, allocation concealment, blinding of participants and personnel, blinding of outcome assessment, incomplete outcome data, selective reporting, and other biases. Each entry could be classified into “low risk”, “unclear” and “high risk” 3 levels. The above results were cross-checked by two independent authors (LYL and WW), and RRW arbitrated the disagreement that existed.

We used the Grading of Recommended Assessment Development and Evaluation (GRADE, Grade Pro version 3.6.1) (26) to assess the quality of the evidence, which was categorized as high, moderate, low, and very low. The GRADE grading reduced the limitations of each study from high quality if any of the following limitations were present (1): design limitations (2); inconsistent results (3); evidence of indirectness (4); imprecision (5); reporting bias.

### Data analysis

Meta-analysis was performed by RevMan version 5.3. Study heterogeneity was assessed as the Cochran Chi-square test and I-square (I^2^) statistic (27, 28). I^2^ < 50% statistics or the P-value>0.01 was defined as low heterogeneity (29). When possible, we used a random-effects model for meta-analysis. For dichotomous variables, a risk ratio (RR) with 95% Confidence Intervals (CIs) was used. The continuous outcomes were represented by the mean difference (MD) of 95% CI. We combined studies that had the same form of acupuncture intervention and the same controls. Due to the small number of included studies and limitations in study quality, we did not perform a subgroup analysis. The funnel plot did not apply to this study because of the small number of included literature (30). For studies whose outcomes could not be combined, we performed a description for individual studies.

## Results

### Search results

**Figure 1:**
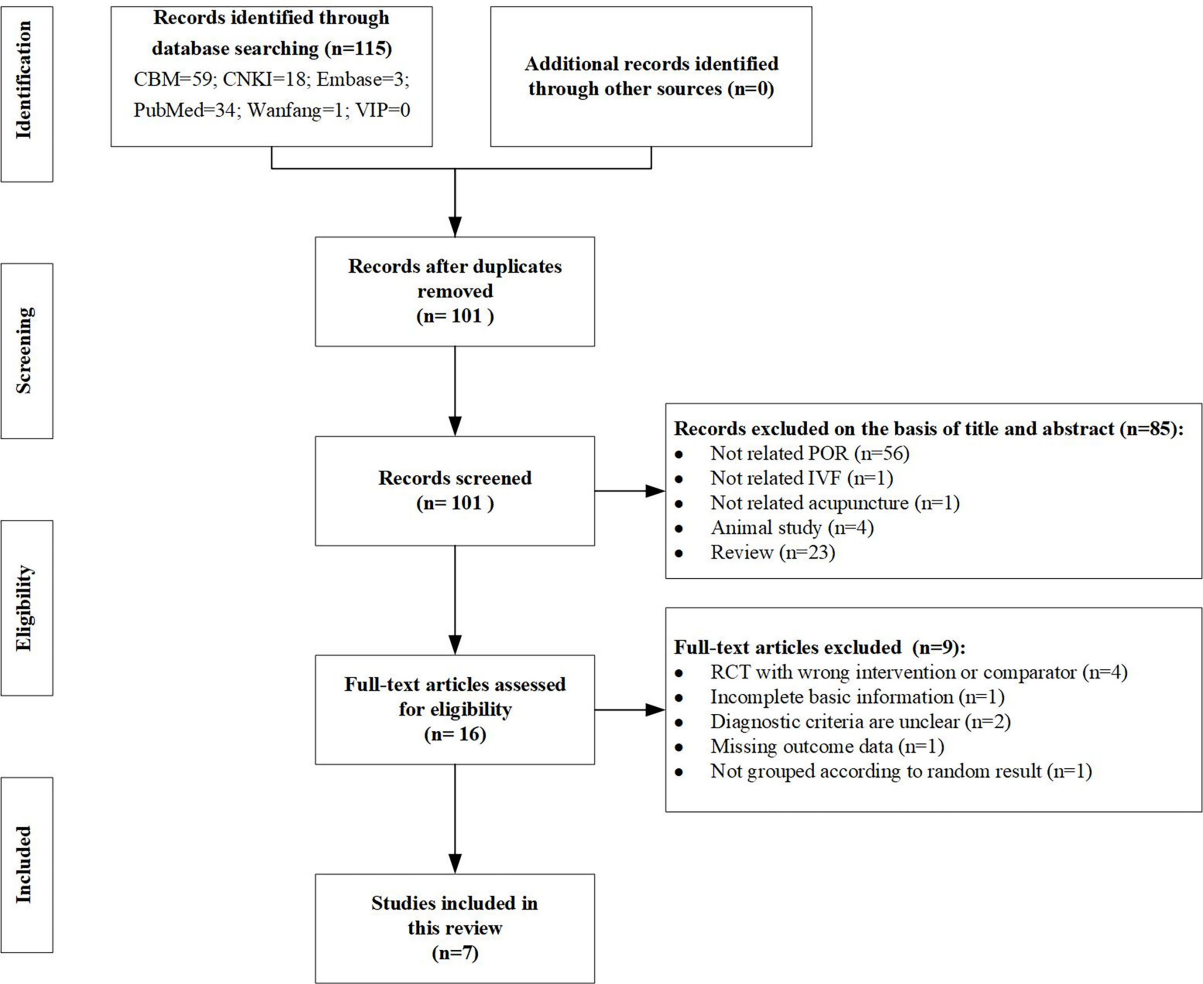
PRISMA flow diagram of the present study.

We identified 115 potentially relevant records from seven databases and four clinical trial registries, of which 101 articles remained after the removal of duplicates. After screening the titles and abstracts, 85 papers were excluded for at least one of the following reasons: case report or review (23 papers), not researching the study of POR (56 papers), not researching the study of IVF (1 paper), animal study (4 papers), and acupuncture was not an intervention (1 paper). By reading the full text, a total of 9 studies were excluded for the following reasons: four with the wrong intervention or comparator (31–34); two with diagnostic criteria unclear (35, 36); one lack of basic information (37); one missing outcome data (38); and one did not group according to random result (39). Ultimately, seven eligible studies remained for this systematic review (40–46). The PRISMA flowchart of the screening process is shown in **Figure 1**.

### Study characteristics

The basic characteristics of the included studies are shown in **Table 1**. The seven included studies were published from 2009 to 2022. A total of 516 participants were enrolled in this review, with sample sizes ranging from 23 to 109 for each study. Six (40, 41, 43–45, 47) studies were conducted in China and only the other study was conducted (42) in South Korea. In two studies (40, 45) were master’s theses, one study (47) was a doctoral dissertation, and the remaining four (41–44) were journal articles. Besides, among these seven articles, six studies (40, 41, 43–45, 47) were published in Chinese, and the other one (42) was published in English. Diagnostic criteria for POR are clear in all seven articles, one article (44) utilized the POR diagnostic of Poseidon, and three articles (42, 45, 47) utilized the POR diagnostic consensus developed by the European Society of Human Reproduction and Embryology (ESHRE) in Bologna, and the clear POR diagnostic consensus of the other three articles (40, 41, 43) was also given.

**Table 1:** Basic information of RCTs included.

Study (year)	Country	Study Design;	Sample Size;	Age (y)	Intervention Group (A); Control Group (B)	Diagnostic criteria	Acupoint	Acupuncture stimulation duration
Yang (2022)	China	RCT	109	(A) 32.06±2.89; (B) 31.40±3.00	(A) Acupuncture plus COH therapy (n=50); (B) COH therapy (n=59)	The Poseidon standard group 3	CV4, KI12, CV6, BL23, CV3, ST36, SP6, LR3, KI3	30min
Zhu (2021)	China	RCT	86	(A) 34.25±3.05; (B) 35.53±3.42	(A) Acupuncture plus IVF (n=42); (B) IVF (n=44)	The Bologna criteria	GV20, LI4, CV4, ST36, SP6, LR3, KI6	30min
Kim (2021)	Korea	RCT;	23;	(A) 37.88±3.87; (B) 40.00±3.83	(A) Acupuncture plus COH therapy (n=12); (B) COH therapy (n=11)	The Bologna criteria	CV3, CV4, EX-CA1, SP6, KI3, SP10, ST36, LR3	NR
Zhou (2019)	China	RCT;	66;	(A) 34.67±4.85; (B) 34.58±4.00	(A) Acupuncture plus COH therapy (n=33); (B) COH therapy (n=33)	The Bologna criteria	(1) GV24, LI4, LR3, CV4, ST36; (2) GV20, BL18, BL20, BL23	30min
Qiu (2012)	China	RCT	92	(A) 29.6; (B) 29.8	(A) TEAS plus COH therapy (n=45); (B) COH therapy (n=47)	Diagnosed POR in (1) or (2) & (3) or (4) (1) Number of oocytes retrieved<5; (2) Number of mature follicles<2~5 on the hCG injection day; (3) Gn used over 17 days and the daily dose of Gn>300IU; (4) The total dose of Gn used in a single ovulation induction cycle≥3500IU	EX-CA1, GV4, BL23, SP6, ST36, CV4, ST25, SP10, GV3	NR
Chen (2011)	China	RCT;	80;	(A) 37.1±5.32; (B) 36.6±4.65	(A) TEAS plus COH therapy (n=40); (B) COH therapy (n=40)	Diagnosed POR in any of the following two (1) Number of bilateral AFC≤5; (2) bFSH≥10IU/L at least twice; or FSH/LH≥3.6; (3) Age≥35 years; (4) History of pelvic surgery	CV4, CV3, SP6, EX-CA1, ST25, BL23, GV3, GV4	30min
Chen (2009)	China	RCT;	60;	(A) 34.33±2.71; (B) 34.60±2.38	(A) Electroacupuncture plus COH therapy (n=30); (B) COH therapy (n=30)	Diagnosed POR in (1) or (2) & (3) or (4) or (5) (1) Serum E_2_<1835 pmol/L on the hCG injection day; (2) Number of AFC≤4; (3) Total dose of exogenous Gn: the total dose of Gn used in a single ovulation induction cycle≥3300U; (4) Daily dose of Gn≥300U; (5) Gn used over 15 days during ovulation induction	CV4, KI3, EX-CA1, SP6, CV3, LR3	30min

RCT, randomized controlled trials; COH, controlled ovarian hyperstimulation; AMH, anti-mullerian hormone; AFC, antral follicle count; MD, mean difference; CI, confidence Intervals; RR, risk ratio; NR, not reported; FSH, follicle-stimulating hormone; LH, luteinizing hormone; E_2_, estradiol; TEAS, transcutaneous electrical acupoint stimulation; CPR, Clinical pregnancy rate; Gn, gonadotropin; POR, poor ovarian response.

As for acupuncture manipulation, manual acupuncture (42, 44, 45, 47), electroacupuncture (41), and TEAS (40, 43) were involved. In addition, the outcomes of the included studies varied, including CPR (40, 41), fertilization rate (40–45, 47), the number of oocytes retrieved per woman (40–45, 47), high-quality embryo rate (40, 41, 43–45, 47), implantation rate (40, 41), the thickness of endometrium (40, 41, 44), cycle cancellation rate (40, 41, 43, 47), FSH (40, 43, 45, 47), LH (40, 41, 43, 45, 47), E_2_ (40, 41, 45), AFC (40, 42, 43), AMH (42), dose of Gn (40, 44, 47), duration of Gn (40, 44, 47), miscarriage (41), etc.

### Quality of studies

**Figure 2:**
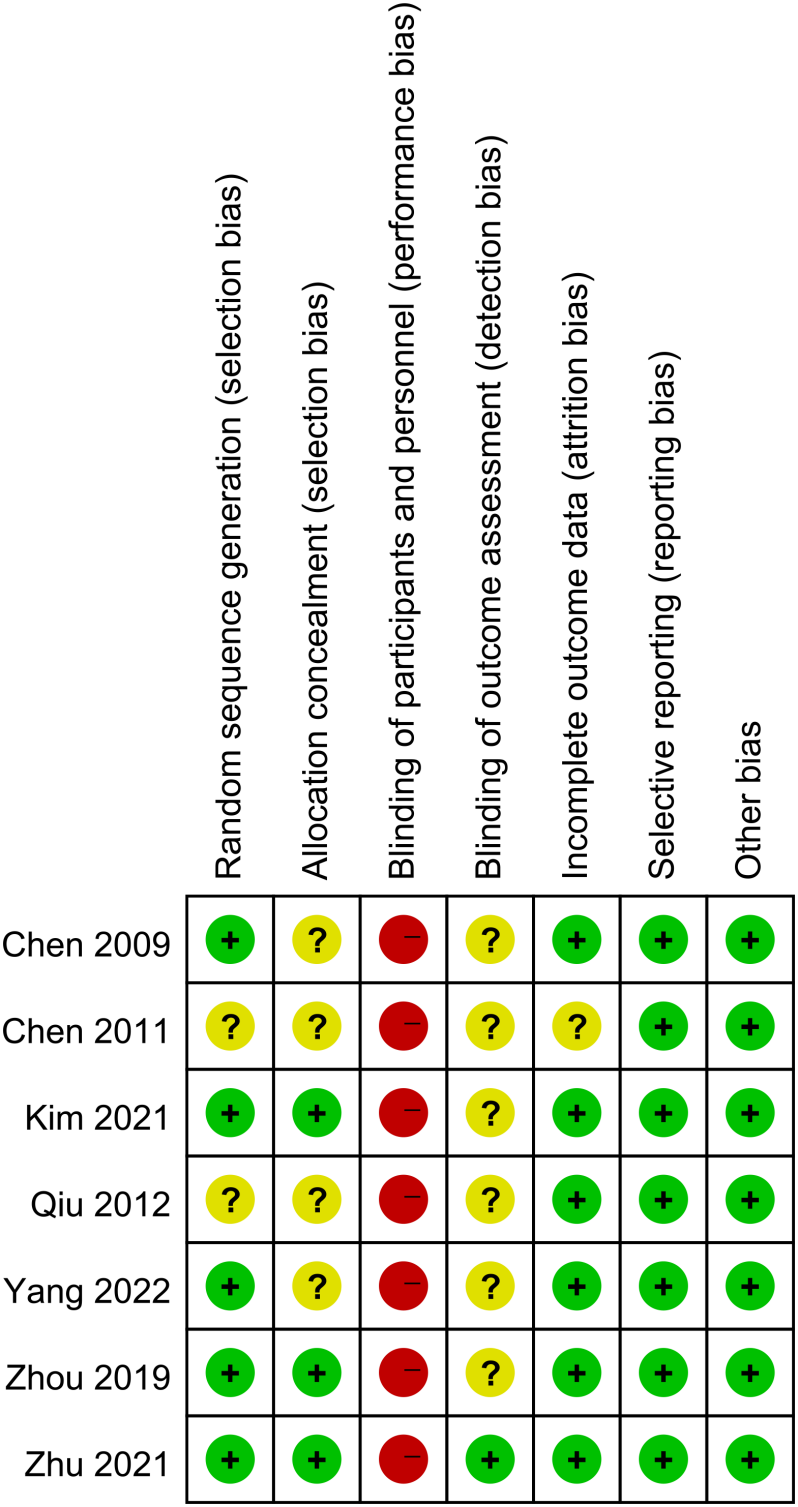
Risk of bias summary.

**Figure 2** demonstrated the risk of bias in each study in this review. The quality of the included studies was generally low or very low. Five studies (41, 42, 44, 45, 47) identified random methods, and another two studies (40, 43) did not mention a specific randomization scheme. Besides, two studies (42, 45) applied allocation concealment. None of the studies mentioned the blinding of participants or investigators. Incomplete outcomes data were deemed to carry a low risk of bias for all the studies except for that by Chen (2011) (40). Outcomes were also not selectively reported in seven studies (40–45, 47). Lastly, seven studies (40–45, 47) were free of other biases. We assessed the evidence of outcome indicators in this meta-analysis to be generally of low or very low quality based on GRADE, owing to the high risk of bias and high heterogeneity. CPR as a primary indicator for evaluating pain was no high-quality evidence. The summary findings of various interventions were conducted (see **Table 2**).

**Table 2:** Quality of evidence based on GRADE.

Outcome Indicators (No. of Comparisons)	Result Summary				
	No. of Participants		Effect Measurement (95% CI)		Quality of Evidence
	Intervention Group	Control Group	Relative Effect	Absolute Effect	
Acupuncture plus COH therapy plus COH therapy					
CPR (2 comparisons of 2 RCTs)	58	54	RR 1.59 (0.80, 3.17)	–	⊕⊕○○ Low ^a, d^
Fertilization rate (7 comparisons of 7 RCTs)	–	–	Not estimate	–	⊕⊕⊕○ Moderate ^a^
Implantation rate (2 comparisons of 2 RCTs)	60	56	RR 2.13 (1.08, 4.21)	–	⊕⊕○○ Low ^a, d^
Cycle cancellation rate (4 comparisons of 4 RCTs)	–	–	Not estimate	–	⊕○○○ Very Low ^a, c, d^
High-quality embryo rate (6 comparisons of 6 RCTs)	–	–	Not estimate	–	⊕⊕○○ Low ^a, d^
Miscarriage rate (1comparisons of 1 RCT)	30	30	RR 0.67 (0.20, 2.26)	–	⊕○○○ Very Low ^a, c, d^
Number of oocytes retrieved (7 comparisons of 7 RCTs)	238	249	–	MD 1.02 (0.72, 1.32)	⊕⊕○○ Low ^a, b^
FSH (4 comparisons of 4 RCTs)	157	164	–	MD -1.52 (-2.41, -0.62)	⊕○○○ Very Low ^a, b, c^
LH (5 comparisons of 5 RCTs)	–	–	–	Not estimate	⊕○○○ Very Low ^a, b, c^
E_2_ (3 comparisons of 3 RCTs)	93	89	–	MD 1667.80 (1578.29, 1757.31)	⊕○○○ Very Low ^a, b, c, d^
AFC (3 comparisons of 3 RCTs)	90	97	–	MD 1.49 (1.04, 1.95)	⊕⊕○○ Low ^a, b^
AMH (1comparisons of 1 RCT)	8	10	–	MD 0.08 (-0.29, 0.45)	⊕⊕○○ Low ^a, d^
Dose of Gn (3 comparisons of 3 RCTs)	122	129	–	MD -19.68 (-164.22, 124.87)	⊕○○○ Very Low ^a, c, d^
Duration of Gn (3 comparisons of 3 RCTs)	122	129	–	MD 0.47 (-0.00, 0.94)	⊕⊕○○ Low ^a, c^
Thickness of endometrium (3 comparisons of 3 RCTs)	110	115	–	MD 0.54 (0.13, 0.96)	⊕⊕○○ Low ^a, c^

a Download one level for serious risk of bias: included studies that did not conduct the blinding method, and unclear risk of bias in one or two domains. Downgraded one level for serious inconsistency: interventions of included studies inconsistent, or the outcome indicators exist statistical heterogeneous. Downgraded one level for serious indirectness: this outcome index cannot directly represent the therapeutic effect of acupuncture on POR patients. Downgraded one level for serious imprecision: very small sample size.

### Results of meta-analysis

#### Primary outcome

CPR was the primary outcome, and it was measured in two trials (40, 41). Acupuncture plus COH therapy could improve CPR, but there was no significant difference compared with COH therapy alone (*p*>0.05, **Figure S2**).

#### Secondary outcome

#### Other pregnancy outcomes

##### Fertilization rate

The fertilization rate of the acupuncture plus COH therapy group and the COH therapy group were compared in the seven trials (40–45, 47). Only Zhou’s study (45) (*p*<0.01) and Chen et al. study (2009) (41) (*p*<0.05) showed that the fertilization rate of the treatment group was significantly higher than that in the control group. Besides, Zhu’s study (47) found that the number of fertilization and the fertilization rate of acupuncture plus IVF group were comparable to those of the IVF group, and the difference was not statistically significant (*p*>0.05). The other four studies (40, 42–44) showed that there was no significant difference in fertilization rate between the intervention group and the control group (*p*>0.05). The specific information is shown in **Table 3**.

**Table 3:** Outcome indicators included in RCTs.

		Yang 2022	Zhu 2021	Kim 2021	Zhou 2019	Qiu 2012	Chen 2011	Chen2009
Primary outcome	CPR (%)						RR 1.71, 95%CI, [0.59, 4.99], *p*>0.05	RR 1.50, 95%CI, [0.61, 3.69], *p*>0.05
Second outcome	Fertilization rate (%)	RR 1.08, 95%CI, [0.96, 1.20], *p*>0.05	RR 1.03, *p*>0.05	RR 0.97, 95%CI, [0.71, 1.31], *p*>0.05	MD 11.32, 95%CI, [0.59, 22.05], *p*<0.05*	RR 1.03, *p*>0.05	RR 0.90, 95%CI, [0.77, 1.05], *p*>0.05	RR 1.18, *p*<0.05*
	Number of oocytes retrieved (n)	MD -0.42, 95%CI, [-1.11, 0.27], *p*>0.05	MD 0.34, 95%CI, [-0.71, 1.39], *p*>0.05	MD 1.45, 95%CI, [-0.08, 2.98], *p*>0.05	MD 1.81, 95%CI, [0.60, 3.02], *p*<0.05*	MD 2.71, 95%CI, [2.12, 3.30], *p*<0.05*	MD 1.09, 95%CI, [0.27, 1.91], *p*<0.05*	MD 0.20, 95%CI, [-0.42, 0.82], *p*>0.05
	High-quality embryos rate (%)	RR 0.98, 95%CI, [0.89, 1.07], *p*>0.05	MD 17.65, 95%CI, [5.74, 29.56], *p*<0.01*		MD 18.99, 95%CI, [3.83, 34.15], *p*<0.05*	RR 1.06, *p*<0.05*	RR 0.89, 95%CI, [0.68, 1.17], *p*>0.05	RR 1.41, *p*<0.05*
	Implantation rate (%)					RR 1.91, 95%CI, [0.63, 5.79], *p*>0.05	RR 2.28, 95%CI, [0.96, 5.44], *p*<0.05*	
	Thickness of endometrium (mm)	MD 0.20, 95%CI, [-0.40, 0.80], *p*>0.05					MD 1.20, 95%CI, [0.40, 2.00], *p*<0.05*	MD 0.05, 95%CI, [-0.03, 0.13], *p*>0.05
	Cycle cancellation rate (%)		MD 0.66, 95%CI, [0.24, 1.84], *p*>0.05			RR 0.62, *p*>0.05	RR 0.60, 95%CI, [0.11, 3.36], *p*>0.05	RR 0.67, 95%CI, [0.12, 3.71], *p*>0.05
Index for reproductive endocrinology	FSH (IU/L)		MD -0.96, 95%CI, [-2.81, 0.89], *p*>0.05		MD -1.79, 95%CI, [-3.36, -0.22], *p*<0.05*	MD -1.05, 95%CI, [-3.00, 0.90], *p*<0.05*	MD -1.79, 95%CI, [-3.36, -0.22]	
	LH (IU/L)		*p*>0.05		MD -0.54, 95%CI, [-2.23, 1.15], *p*>0.05	MD 0.00, 95%CI, [-0.84, 0.84], *p*>0.05	MD -0.86, 95%CI, [-2.43, 0.71], *p*>0.05	MD 0.13, 95%CI, [-0.19, 0.45], *p*>0.05
	E_2_ (pmol/L)				MD 1678.71, 95%CI, [1588.40, 1769.02], p<0.05*		MD 2176.74, 95%CI, [190.66, 4162.82], *p*<0.05*	MD 915.53, 95%CI, [199.50, 1631.56], *p*<0.05*
	AFC (n)			MD -0.47, 95%CI, [-2.44, 1.50], *p*>0.05		MD 1.76, 95%CI, [1.23, 2.29], *p*<0.05*	MD 1.08, 95%CI, [0.10, 2.06], *p*>0.05	
	AMH (ng/mL)			MD 0.08, 95%CI, [-0.29, 0.45], *p*>0.05				
	Dose of Gn (IU)	MD -24.78, 95%CI, [-191.42, 141.86], *p*>0.05	MD -20.37, 95%CI, [-434.37, 393.63], *p*>0.05				MD 11.55, 95%CI, [-396.11, 419.21], *p*>0.05	
	Duration of Gn (day)	MD 0.61, 95%CI, [-0.04, 1.26], *p*>0.05	MD -0.03, 95%CI, [-1.35, 1.29], *p*>0.05				MD 0.44, 95%CI, [-0.36, 1.24], *p*>0.05	
Adverse effect	Miscarriage rate (%)							RR 0.67, 95%CI, [0.20, 2.26], *p*>0.05

CPR, Clinical pregnancy rate; FSH, follicle-stimulating hormone; LH, luteinizing hormone; E_2_, estradiol; AMH, anti-mullerian hormone; AFC, antral follicle count; Gn, gonadotropin; MD, mean difference; CI, confidence Intervals; RR, risk ratio.

##### Number of oocytes retrieved

**Figure 3:**
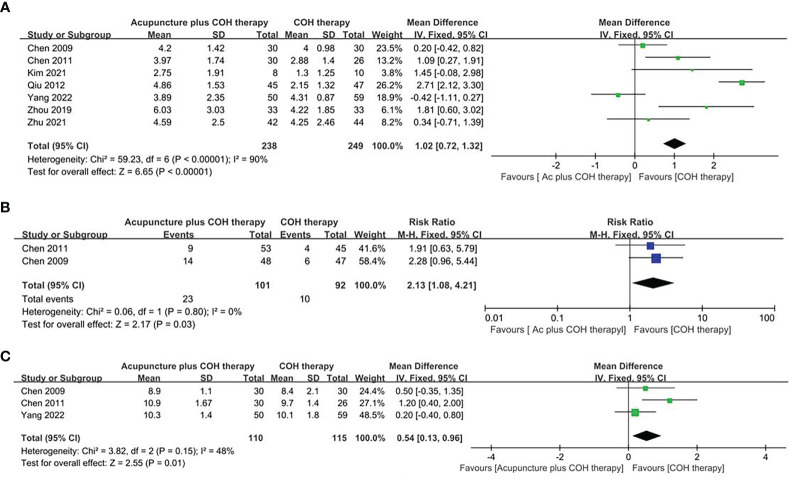
Forest plot of the acupuncture plus controlled ovarian hyperstimulation (COH) therapy and the COH therapy. **(A)** Forest plot for the number of oocytes retrieved of random effect model. **(B)** Forest plot for the implantation rate of random effect model. **(C)** Forest plot for the thickness of endometrium of random effect model.

The number of oocytes retrieved in the acupuncture plus COH therapy group and COH therapy group was recorded in seven studies (40–45, 47). Meta-analysis implied that the number of oocytes retrieved was significantly higher in the acupuncture group (MD=1.02, 95%CI [0.72, 1.32], *p*<0.00001, **Figure 3A**). The I^2^ of this result showed high heterogeneity. A sensitivity analysis was conducted, in which one study at a time was removed and the others analyzed to estimate whether the results could have been affected markedly by a single time. The analysis showed the stability of the result.

##### High-quality embryo rate

Six studies (40, 41, 43–45, 47) reported high-quality embryo rates. A total of 6 studies evaluated 469 registered participants. Two studies (40, 44) showed no significant difference between the intervention group and the control group. The remaining four studies (41, 43, 45, 47) showed that the high-quality embryo rate of the intervention group was significantly higher than that of the control group. The specific information is shown in **Table 3**.

##### Implantation rate

Two studies (40, 41) reported the implantation rate of the acupuncture plus COH therapy group and the COH therapy group. The random effect model showed that the implantation rate of the acupuncture plus COH therapy group was higher than that of the COH therapy group (RR=2.13, 95%CI [1.08, 4.21], *p*=0.03, **Figure 3B**), and the results were statistically significant between the two studies.

##### Thickness of endometrium

Three studies (40, 41, 44) compared the thickness of endometrium between the acupuncture plus COH therapy group and the COH therapy group. The random effect model showed that the thickness of endometrial of the acupuncture plus COH therapy group was significantly higher than that of the COH therapy group (MD=0.54, 95%CI [0.13, 0.96], *p*=0.01, **Figure 3C**).

##### Cycle cancellation rate

Four studies (40, 41, 43, 47) reported the cycle cancellation rates between the two groups. Compared with the control group, the cycle cancellation rate of the intervention group had a decreasing trend, but the difference was not statistically significant in these four studies (40, 41, 43, 47) (*p*>0.05, **Table 3**).

### Index for reproductive endocrinology

#### FSH

**Figure 4:**
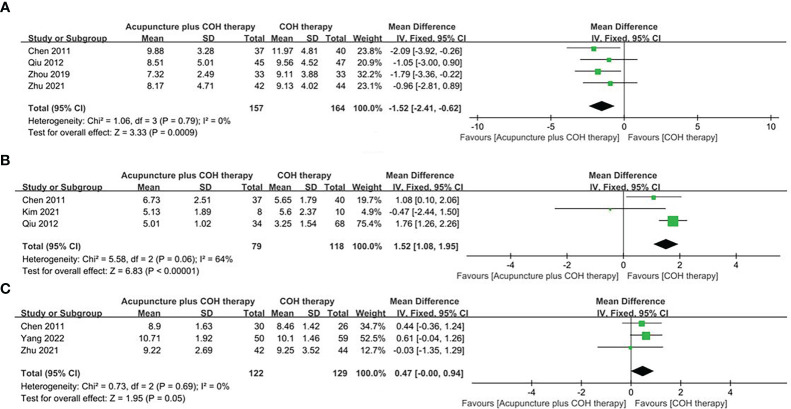
Forest plot of the acupuncture plus controlled ovarian hyperstimulation (COH) therapy and the COH therapy. **(A)** Forest plot for FSH of random effect model. **(B)** Forest plot for AFC of random effect model. **(C)** Forest plot for the duration of Gn of random effect model.

Four studies (40, 43, 45, 47) reported FSH values of 321 participants in the acupuncture plus COH therapy group and the COH therapy group. The random effect model showed that the FSH value of the acupuncture plus COH therapy group was significantly lower than that of the COH therapy group (MD=-1.52, 95%CI [-2.41, -0.62], *p*=0.0009, **Figure 4A**).

#### LH

Five studies (40, 41, 43, 45, 47) reported LH values in the acupuncture plus COH therapy group and the COH therapy group. Five studies (40, 41, 43, 45, 47) all showed no significant difference in the regulation of LH between the intervention group and the control group (*p*>0.05). The specific information is shown in **Table 3**.

#### E_2_

E_2_ value was reported in 3 studies (40, 41, 45). Meta-analysis results showed that the E_2_ of the acupuncture plus COH therapy group was significantly higher than that of the COH therapy group (MD=1667.80, 95%CI [1578.29, 1757.31], *p*<0.00001, **Figure S3**).

#### AFC

Three studies (40, 42, 43) reported AFC in both groups. The random effect model showed that the AFC in the acupuncture plus COH therapy group was significantly higher than that in the COH therapy group (MD=1.49, 95%CI [1.04, 1.95], *p*<0.00001, **Figure 4B**). Besides, it was found that Kim et al. study (42) caused high heterogeneity after sensitivity analysis.

#### AMH

Only one study (42) reported the AMH values of the acupuncture plus COH therapy group and the COH therapy group. The random effects model showed no significant difference in AMH levels between the two groups (*p*>0.05, **Table 3**).

#### Dose and duration of Gn

Three studies (40, 44, 47) reported the dose and duration of Gn in then acupuncture plus COH therapy and COH therapy groups. The random effects model showed no significant difference in the dose of Gn between the two groups (MD=-19.68, 95%CI [-164.224, 124.87], *p*>0.05, **Figure S4** **)**. But the duration of Gn in the intervention group was significantly higher than that in the control group (MD=0.47, 95%CI [0.00, 0.94], *p*=0.05, **Figure 4C**).

### Adverse effect

#### Miscarriage rate

Only one study (41) reported miscarriage rates in the acupuncture plus COH therapy group and the COH therapy group. The results showed that there was no difference in the miscarriage rate between the two groups (*p*>0.05, **Table 3**).

## Discussion

This review included 7 studies (516 POR patients) and investigated the effectiveness of acupuncture on patients with POR through meta-analysis. Compared with the previous systematic review (22), we updated the dates of relevant studies and added outcome indicators. Finally, 7 different studies (40–46) were included. Due to the limitations of treatment methods, there was no literature reporting blinding, resulting in higher heterogeneity. This study found that acupuncture could improve the ovarian function of POR patients to a certain extent. The evidence grade for the outcome indicators of this study was mostly low or very low. Due to the limitations of treatment methods, it was not clear whether allocation concealment is adopted during the research process, inconsistent intervention measures taken in the included studies, and too few samples lead to higher heterogeneity and ultimately lower evidence levels.

A previous study (36) indicated that electroacupuncture before transplantation could significantly improve CPR, which is different from our results. We found that acupuncture treatment could not improve the CPR between the two groups (RR=1.59, 95%CI [0.80, 3.17], *p*=0.19, **Figure S2**). It might because CPR was reported in only two studies (40, 41), resulting in too few samples (n=112) to produce convincing results. Our meta-analysis also found acupuncture could increase the number of oocytes retrieved by POR women undergoing IVF, and increase the implantation rate and the thickness of endometrium, to prepare for the successful implantation of fertilized oocytes. And Lian et al. (48) confirmed that acupuncture can increase the number of retrieved oocytes in POR women, improve fertilization rate, and promote local blood circulation of the uterus and ovary, thereby improving clinical efficacy, which is consistent with our findings. However, whether acupuncture is effective for POR women’s fertilization rate and high-quality embryo rate has not yet been clarified. For this result, expanding the sample size and setting up more rigorous experiments for further research is suggested.

Regarding sex hormones, our results showed acupuncture could considerably decrease FSH levels. Research (39) found that acupuncture could significantly reduce FSH levels in patients with POR and positively regulate basal endocrine levels. Besides, studies (14, 18, 19, 49) found acupuncture could activate aromatase, benign adjust the reproductive axis, promote the generation of neuropeptide Y, improve the local blood flow of the ovary, and improve the utilization efficiency of estrogen, which also pointed out that acupuncture improved the reproductive system by inhibiting the increase of FSH and LH to improve ovarian response as well.

In addition, the results of this study showed that the acupuncture group could significantly increase AFC, but did not have any advantages in improving AMH. Studies (50, 51) have shown that AMH and AFC are the most reliable and accurate predictors of POR, which can reflect the ovarian reserve of POR women. The decrease of AFC in adult women might be related to age (52), race (53–55), genetic (56), and smoking (57) etc. And the decrease in the reserve function of the ovaries was characterized by a decrease in pregnancy rate, an increase in the rate of miscarriage, and a decrease in the rate of simultaneous live births (58). Therefore, it was necessary to find a treatment that can increase AFC. Studies (59–61) have shown that acupuncture, traditional Chinese medicine or acupuncture combined with medicine can effectively improve AFC, thereby improving ovarian reserve function.

Before 2010, there were no clear diagnostic criteria for POR internationally. In 2011, the ESHRE and the American Society for Reproductive Medicine (ASRM) discussed and formulated the Bologna Consensus of POR (1), which is currently widely accepted diagnostic criteria in most countries. In 2016, the POSEIDON Group proposed a new POR standard, the Poseidon Standard (12). However, only three researches (42, 45, 46) included in this study used the Bologna Consensus in this study, one study (44) used POSIDON diagnostic, resulting in differences in the diagnostic criteria of POR, which may affect high heterogeneity. Therefore, it is recommended that more studies use uniform diagnostic criteria to reduce the differences between studies. Besides, there are studies also confirming that advanced age (9, 62) is an important factor causing infertility in POR women. Some studies (63, 64) only use AMH as the prediction of ovarian function, but since AMH peaked during puberty and then decreased with age, its change is closely related to age. Therefore, we believe that the combination of age and the AMH may have a better predictive value. It is recommended that more studies use uniform diagnostic criteria to reduce the heterogeneity between studies and improve the evidence level of the results.

Different acupuncture strategies, acupuncture time points, and acupuncture frequency might be important factors affecting the results as well. Studies have shown that increasing the number of acupuncture can improve uterine blood flow (65), increase serum cortisol and prolactin (66), and improve IVF pregnancy outcomes (67). Wang et al.’s (68) research also confirmed that acupuncture at different time points would produce different IVF clinical pregnancy results. But for the acupuncture treatment plan, timing, frequency, and other aspects of POR, there is still no consensus. Therefore, in clinical trials, these factors that affect should be paid attention to and differences between studies should be reduced.

## Limitations and conclusion

In summary, acupuncture is a safe and effective method to improve POR and has great potential in regulating sex hormone levels, and improving ovarian reserve. However, the heterogeneity among the included studies had an impact on the results, especially in acupuncture treatment, acupoint selection and acupuncture techniques. Some studies did not follow up the patients for a long time, and did not mention indicators such as PCR and live birth rate, which made it impossible to make statistics. In the future, more relevant variables need to be added to improve the data, verify the effectiveness of acupuncture in the treatment of POR, and provide more possibilities and references for future clinical research and practice.

## Data availability statement

The original contributions presented in the study are included in the article/**Supplementary Material**. Further inquiries can be directed to the corresponding authors.

## Author contributions

R-RW conceived, designed, and drafted the manuscript. L-YL, M-HS, and WW performed data acquisition and quality control. Y-YL, X-LG, DG, and YZ performed data preparation. X-YZ and HY performed the statistical analysis, and S-YY, F-RL, and JY provided guidance and critical revisions to the manuscript. All authors contributed to the article and approved the submitted version.

## References

[1] Ferraretti AP La Marca A Fauser BC Tarlatzis B Nargund G Gianaroli L . ESHRE consensus on the definition of ‘poor response’ to ovarian stimulation for *in vitro* fertilization: the Bologna criteria. Hum Reprod (Oxford England). (2011) 26(7):1616–24. 10.1093/humrep/der092 21505041

[2] Alviggi C Conforti A Esteves SC Vallone R Venturella R Staiano S . Understanding ovarian hypo-response to exogenous gonadotropin in ovarian stimulation and its new proposed marker-the follicle-To-Oocyte (FOI) index. Front endocrinology. (2018) 9:589. 10.3389/fendo.2018.00589 PMC6199413 30386293

[3] Polyzos NP Devroey P . A systematic review of randomized trials for the treatment of poor ovarian responders: Is there any light at the end of the tunnel? Fertility sterility (2011) 96(5):1058–61.e7. 22036048 10.1016/j.fertnstert.2011.09.048

[4] Zhang R Feng XJ Guan Q Cui W Zheng Y Sun W . Increase of success rate for women undergoing embryo transfer by transcutaneous electrical acupoint stimulation: A prospective randomized placebo-controlled study. Fertility sterility. (2011) 96(4):912–6. 10.1016/j.fertnstert.2011.07.1093 21862001

[5] Tarlatzis BC Zepiridis L Grimbizis G Bontis J . Clinical management of low ovarian response to stimulation for IVF: A systematic review. Hum Reprod update. (2003) 9(1):61–76. 12638782 10.1093/humupd/dmg007

[6] Wu XQ Kong R Tian L Gong F Hu LL Sun YP . A consensus of poor ovarian response. J Reprod Contraception. (2015) 35(2):71–9.

[7] de Vet A Laven JS de Jong FH Themmen AP Fauser BC . Antimüllerian hormone serum levels: A putative marker for ovarian aging. Fertility sterility. (2002) 77(2):357–62. 10.1016/s0015-0282(01)02993-4 11821097

[8] Al-Inany H Aboulghar MA Mansour RT Serour GI . Optimizing GnRH antagonist administration: Meta-analysis of fixed versus flexible protocol. Reprod biomedicine online. (2005) 10(5):567–70. 10.1016/s1472-6483(10)61661-6 15949209

[9] Wang X Jin L Mao YD Shi JZ Huang R Jiang YN . Evaluation of ovarian reserve tests and age in the prediction of poor ovarian response to controlled ovarian stimulation-a real-world data analysis of 89,002 patients. Front endocrinology. (2021) 12:702061. 10.3389/fendo.2021.702061 PMC8435745 34526967

[10] Alviggi C Clarizia R Pettersson K Mollo A Humaidan P Strina I . Suboptimal response to GnRHa long protocol is associated with a common LH polymorphism. Reprod biomedicine online. (2011) 22 Suppl 1:S67–72. 10.1016/S1472-6483(11)60011-4 21575852

[11] Nargund G Cheng WC Parsons J . The impact of ovarian cystectomy on ovarian response to stimulation during *in-vitro* fertilization cycles. Hum Reprod (Oxford England). (1996) 11(1):81–3. 10.1093/oxfordjournals.humrep.a019043 8671163

[12] Alviggi C Andersen CY Buehler K Conforti A De Placido G Esteves SC . A new more detailed stratification of low responders to ovarian stimulation: from a poor ovarian response to a low prognosis concept. Fertility sterility. (2016) 105(6):1452–3. 10.1016/j.fertnstert.2016.02.005 26921622

[13] Çelik G Sütçü HK Akpak YK Akar ME . A flexible multidose GnRH antagonist versus a microdose flare-up GnRH agonist combined with a flexible multidose GnRH antagonist protocol in poor responders to IVF. BioMed Res Int (2015) 2015:970163. 26161425 10.1155/2015/970163 PMC4487334

[14] Qu F Li R Sun W Lin G Zhang R Yang J . Use of electroacupuncture and transcutaneous electrical acupoint stimulation in reproductive medicine: A group consensus. J Zhejiang Univ Sci B (2017) 18(3):186–93. 10.1631/jzus.B1600437 PMC5369245 28271655

[15] Ng EH So WS Gao J Wong YY Ho PC . The role of acupuncture in the management of subfertility. Fertility sterility. (2008) 90(1):1–13. 18440533 10.1016/j.fertnstert.2008.02.094

[16] Zheng CH Huang GY Zhang MM Wang W . Effects of acupuncture on pregnancy rates in women undergoing *in vitro* fertilization: A systematic review and meta-analysis. Fertil Steril. (2012) 97(3):599–611. 22243605 10.1016/j.fertnstert.2011.12.007

[17] Zhou L Xia Y Ma X Tang L Lu J Tang Q . [Effects of “menstrual cycle-based acupuncture therapy” on IVF-ET in patients with decline in ovarian reserve]. Zhongguo zhen jiu = Chin acupuncture moxibustion. (2016) 36(1):25–8. 26946729

[18] Mu Y Li Q Cheng J Shen J Jin X Xie Z . Integrated miRNA-seq analysis reveals the molecular mechanism underlying the effect of acupuncture on endometrial receptivity in patients undergoing fertilization: embryo transplantation. 3 Biotech. (2020) 10(1):6. 10.1007/s13205-019-1990-3 PMC6879696 31824817

[19] Wang Q Lu G Xie ZJ Li HX Shen MH . [Effect of moxibustion on Nrf2/HO-1 signaling pathway in rats with diminished ovarian reserve]. Zhongguo zhen jiu = Chin acupuncture moxibustion. (2021) 41(1):53–8. 10.13703/j.0255-2930.20191128-k0005 33559443

[20] Fan S Fang YG . Research progress of acupuncture for the improvement of ovarian reserve by regulating different signal pathways. Zhen ci yan jiu = Acupuncture Res (2022) 47(7):1–8. 10.13702/j.1000-0607.20210330 35880284

[21] Mi H Gong AL Sun W Feng XH Li Y . Therapeutic effect of transcutaneous electrical acupoint stimulation on 30 cases of low ovarian response. J Shandong Univ TCM. (2013) 37(06):495–6.

[22] Jang S Kim KH Jun JH You S . Acupuncture for *in vitro* fertilization in women with poor ovarian response: a systematic review. Integr Med Res (2020) 9(2):100395. 32322482 10.1016/j.imr.2020.02.003 PMC7160570

[23] Shen C Wu M Shu D Zhao X Gao Y . The role of acupuncture in *in vitro* fertilization: A systematic review and meta-analysis. Gynecologic obstetric Invest (2015) 79(1):1–12. 10.1159/000362231 24854767

[24] Page MJ McKenzie JE Bossuyt PM Boutron I Hoffmann TC Mulrow CD . PRISMA 2020 statement: An updated guideline for reporting systematic reviews. BMJ (Clinical Res ed) (2021) 372:n71. 10.1136/bmj.n71 PMC8005924 33782057

[25] Cumpston M Li T Page MJ Chandler J Welch VA Higgins JP . Updated guidance for trusted systematic reviews: A new edition of the cochrane handbook for systematic reviews of interventions. Cochrane Database systematic Rev (2019) 10:Ed000142. 10.1002/14651858.ED000142 PMC10284251 31643080

[26] Guyatt GH Oxman AD Vist GE Kunz R Falck-Ytter Y Alonso-Coello P . GRADE: an emerging consensus on rating quality of evidence and strength of recommendations. BMJ (Clinical Res ed). (2008) 336(7650):924–6. 10.1136/bmj.39489.470347.AD PMC2335261 18436948

[27] Bohning D Lerdsuwansri R Holling H . Some general points on the -measure of heterogeneity in meta-analysis. Metrika (2017) 80(6-8):685–95.

[28] Higgins JP Thompson SG Deeks JJ Altman DG . Measuring inconsistency in meta-analyses. BMJ (Clinical Res ed). (2003) 327(7414):557–60. 10.1136/bmj.327.7414.557 PMC192859 12958120

[29] Guyatt GH Oxman AD Kunz R Woodcock J Brozek J Helfand M . GRADE guidelines: 7. rating the quality of evidence–inconsistency. J Clin Epidemiol (2011) 64(12):1294–302. 10.1016/j.jclinepi.2011.03.017 21803546

[30] Sterne JA Sutton AJ Ioannidis JP Terrin N Jones DR Lau J . Recommendations for examining and interpreting funnel plot asymmetry in meta-analyses of randomised controlled trials. BMJ (Clinical Res ed). (2011) 343:d4002. 10.1136/bmj.d4002 21784880

[31] He WS . Clinical study on improving the quality of IVF oocytes by different time of acupuncture intervention [Master’s thesis]: Chengdu university of traditional Chinese medicine. (2020). doi: 10.26988/d.cnki.gcdzu.2020.000394

[32] Jiang GL Wan P An XQ Yu WT Wang P Zhou XM . Efficacy of supplemented erxian decoction combined with acupoint application for poor ovarian response. J Physiol pharmacology: an Off J Polish Physiol Soc (2020) 71(2):249–55. 10.26402/jpp.2020.2.09 32776907

[33] Mi H . Study of the effect of transcutanclus electrical acupoint stimulation (TEAS) to the patients with ovarian low response in the effect of pregnancy outcome during in-vitro fertilization-embryo transfer (IVF-ET) cycle [Master’s thesis]: Shandong university of traditional Chinese medicine. (2014).

[34] Zheng Y Feng X Mi H Yao Y Zhao Y Li J . Effects of transcutaneous electrical acupoint stimulation on ovarian reserve of patients with diminished ovarian reserve in *in vitro* fertilization and embryo transfer cycles. J obstetrics gynaecology Res (2015) 41(12):1905–11. 10.1111/jog.12810 26455718

[35] Gao XA Chen MZ Ma WM Luo GQ Pan JL . The clinical effect of acupuncture combined with traditional Chinese medicine (CM) artificial cycle therapy on the treatment of *in vitro* fertilization (IVF) cycle with poor ovarian response. J Foshan University(Natural Sci Edition). (2017) 35(06):57–61.

[36] Shen J Gao YL Lu G Chen L Cheng J Xia YB . [Effect of electroacupuncture on endometrial receptivity and IVF-ET pregnancy outcomes in patients with diminished ovarian reserve]. Zhongguo zhen jiu = Chin acupuncture moxibustion. (2022) 42(8):879–83. 10.13703/j.0255-2930.20210901-k0002 35938330

[37] Zhang XY Zhang XX Wang L Wang J Wang KK Zhao SQ . Effect of acupuncture combined with kuntai capsuleson the pregnancy outcome of infertile patients with hyporesponsive ovarian failure in IVF-ET. Chin J Hum Sexuality. (2018) 27(11):68–71.

[38] Dong XL Ran JK Zhang HJ Chen K Li HX . Acupuncture combined with medication improves endocrine hormone levels and ovarian reserve function in poor ovarian response patients undergoing *in vitro* fertilization-embryo transplantation. Zhen ci yan jiu = Acupuncture Res (2019) 44(8):599–604. 10.13702/j.1000-0607.180779 31475495

[39] Zong DK . Study on the effect and mechanism of bu shen tiao chong in acupuncture on IVF treatment for patients with diminished ovarian reserve *[Master’s thesis]*: Shanghai university of traditional Chinese medicine;. (2020). doi: 10.27320/d.cnki.gszyu.2020.000375

[40] Chen C . Study of the effect of transcutanclus electrical acupoint stimulation intervene in the patients with ovarian poor respond [Master’s thesis]: Shandong university of traditional Chinese medicine;. (2011).

[41] Chen J Liu LL Cui W Cui W . Effects of electroacupuncture on *in vitro* fertilization-embryo transfer (IVF-ET) of patients with poor ovarian response. Zhongguo zhen jiu = Chin acupuncture moxibustion. (2009) 29(10):775–9. 19873910

[42] Kim J Lee H Choi TY Kim JI Kang BK Lee MS . Acupuncture for poor ovarian response: A randomized controlled trial. J Clin Med (2021) 10(10):2182–2182. 10.3390/jcm10102182 PMC8158119 34070086

[43] Qiu WX Zhang XY Lin XX Chen WJ Zhang B . Effect of transcutaneous acupoint electrical stimulation on embryo quality and pregnancy outcome in low ovarian reaction patients. Chin J Hum Sexuality. (2012) 21(7):22–4.

[44] Yang T Gou WJ Wang W Zhao C Chen XL Ma XL . Effect of traditional acupuncture on IVF-ET adjuvant therapy and cytokines in POR patients in poseidon group 3: a randomized controlled study. J Reprod Med (2022) 31(11):1513–9.

[45] Zhou T . Effect of acupuncture on the number of retrieved oocytes and the rate of high-grade embryo in patients with poor ovarian response to IVF mild-stimulation [Master’s thesis]: Chengdu university of traditional Chinese medicine;. (2019).

[46] Zhu XY . Study of acupuncture improving the oocytes quality of poor ovarian respond patients in IVF based on DNA methylation [doctoral thesis]: Chengdu university of traditional Chinese medicine;. (2021). doi: 10.26988/d.cnki.gcdzu.2021.000023

[47] Zhou X Wu Q Liu M Zhu W Ren Q Wang Y . Moxibustion for essential hypertension and hypertensive symptoms: A systematic review of 18 randomized controlled trials. Complementary Med Res (2021) 28(5):435–45. 10.1159/000513701 33494086

[48] Lian F Li R . Treatment of poor ovarian response with shen deficiency by transcutaneous acupoint electrical stimulation combined intracavitary physiotherapy. Chin J Integrated Traditional Western Med (2017) 37(05):522–5.

[49] Li Y Xia Y liu SM Ju ZY Shi XL Chen MG . Clinical study on electroacupuncture for perimenopause syndrome. J Acupuncture Tuina Science. (2011) 9(05):278–82.

[50] La Marca A Sunkara SK . Individualization of controlled ovarian stimulation in IVF using ovarian reserve markers: from theory to practice. Hum Reprod update. (2014) 20(1):124–40. 10.1093/humupd/dmt037 24077980

[51] Silberstein T MacLaughlin DT Shai I Trimarchi JR Lambert-Messerlian G Seifer DB . Mullerian inhibiting substance levels at the time of HCG administration in IVF cycles predict both ovarian reserve and embryo morphology. Hum Reprod (Oxford England). (2006) 21(1):159–63. 10.1093/humrep/dei270 16123085

[52] Wiser A Shalom-Paz E Hyman JH Sokal-Arnon T Bantan N Holzer H . Age-related normogram for antral follicle count in women with polycystic ovary syndrome. Reprod biomedicine online. (2013) 27(4):414–8. 10.1016/j.rbmo.2013.06.016 23948452

[53] Melado L Vitorino R Coughlan C Bixio LD Arnanz A Elkhatib I . Ethnic and sociocultural differences in ovarian reserve: Age-specific anti-müllerian hormone values and antral follicle count for women of the Arabian peninsula. Front endocrinology. (2021) 12:735116. 10.3389/fendo.2021.735116 PMC8567992 34745004

[54] Schuh SM Kadie J Rosen MP Sternfeld B Reijo Pera RA Cedars MI . Links between age at menarche, antral follicle count, and body mass index in African American and European American women. Fertility sterility. (2019) 111(1):122–31. 10.1016/j.fertnstert.2018.09.007 30611402

[55] Loy SL Cheung YB Fortier MV Ong CL Tan HH Nadarajah S . Age-related nomograms for antral follicle count and anti-mullerian hormone for subfertile Chinese women in Singapore. PloS One (2017) 12(12):e0189830. 29240820 10.1371/journal.pone.0189830 PMC5730199

[56] Rosen MP Johnstone EB Gillham SJ Modan AE Lipshutz AK Reijo-Pera R . Is antral follicle count a genetic trait? Menopause (New York NY) (2010) 17(1):109–13. 10.1097/gme.0b013e3181b48a88 19752762

[57] Bhide P Timlick E Kulkarni A Gudi A Shah A Homburg R . Effect of cigarette smoking on serum anti-mullerian hormone and antral follicle count in women seeking fertility treatment: A prospective cross-sectional study. BMJ Open (2022) 12(3):e049646. 10.1136/bmjopen-2021-049646 PMC8971761 35361635

[58] Kimberly L Case A Cheung AP Sierra S AlAsiri S Carranza-Mamane B . Advanced reproductive age and fertility: no. 269, November 2011. Int J gynaecology obstetrics: Off Organ Int Fed Gynaecology Obstetrics. (2012) 117(1):95–102. 10.1016/j.ijgo.2011.11.002 22506284

[59] Zhang Y . Clinical effect of acupuncture on patients with premature ovarian insufficiency and its influence on intestinal flora [Master’s thesis]: Nanjing university of Chinese medicine;. (2022). doi: 10.27253/d.cnki.gnjzu.2022.000184

[60] Sun HY Sun L Shi JF Liu CL Liang ZC Yang H . Observation of curative effect on premature ovarian insufficiency based on syndrome differentiation of blood stasis and heat. Modern J Integrated Traditional Chin Western Med (2022) 31(02):174–8+97.

[61] Zhu XL Sun Y Ma DZ Hu XX Qian YQ Zhang H . Observation on bubo decoction combined with acupuncture in treatment of diminished ovarian reserve. Chinese archives of traditional Chinese medicine (2021) 39(11):247–50.

[62] Cohen Y Tannus S Alzawawi N Son WY Dahan M Buckett W . Poor ovarian response as a predictor for live birth in older women undergoing IVF. Reprod biomedicine online. (2018) 36(4):435–41. 10.1016/j.rbmo.2018.01.008 29478839

[63] Grynnerup AG Lindhard A Sørensen S . The role of anti-müllerian hormone in female fertility and infertility - an overview. Acta obstetricia gynecologica Scandinavica. (2012) 91(11):1252–60. 10.1111/j.1600-0412.2012.01471.x 22646322

[64] Moolhuijsen LME Visser JA . Anti-müllerian hormone and ovarian reserve: Update on assessing ovarian function. J Clin Endocrinol Metab (2020) 105(11):3361–73. 10.1210/clinem/dgaa513 PMC7486884 32770239

[65] Stener-Victorin E Jedel E Janson PO Sverrisdottir YB . Low-frequency electroacupuncture and physical exercise decrease high muscle sympathetic nerve activity in polycystic ovary syndrome. Am J Physiol Regulatory Integr Comp Physiol (2009) 297(2):R387–95. 10.1152/ajpregu.00197.2009 19494176

[66] Magarelli PC Cridennda DK Cohen M . Changes in serum cortisol and prolactin associated with acupuncture during controlled ovarian hyperstimulation in women undergoing *in vitro* fertilization-embryo transfer treatment. Fertil Steril. (2009) 92(6):1870–9. 10.1016/j.fertnstert.2008.10.067 19118825

[67] Hullender Rubin LE Opsahl MS Wiemer KE Mist SD Caughey AB . Impact of whole systems traditional Chinese medicine on *in-vitro* fertilization outcomes. Reprod biomedicine online. (2015) 30(6):602–12. 10.1016/j.rbmo.2015.02.005 PMC4458185 25911598

[68] Wang YR . Effect of acupuncture intervention at different time points on the pregnancy rate of *in vitro* fertilization-frozen embryo transfer *[Doctor’s thesis]*: Beijing university of Chinese medicine;. (2021). doi: 10.26973/d.cnki.gbjzu.2021.000114 36075593

