# Supplementary material for: Systematic review of acupuncture to improve ovarian function in women with poor ovarian response
Source: Front Endocrinol (Lausanne). 2023 Mar 13;14:1028853. doi: 10.3389/fendo.2023.1028853 (PMC10040749; doi:10.3389/fendo.2023.1028853)
Supplement: Supplementary file 1 [file DataSheet_1.doc]

# Search strategy

**1. PubMed**

**Search Date: January 30, 2023**

(((("Acupuncture" OR "Acupuncture Therapy"[MeSH Major Topic]) OR ("Transcutaneous Electric Nerve Stimulation")) OR ((acupunct*[Title/Abstract] OR acupress*[Title/Abstract] OR acupoint*[Title/Abstract] OR electroacupunt*[Title/Abstract] OR electro-acupunt*[Title/Abstract] OR moxibust*[Title/Abstract] OR point injection[Title/Abstract] OR transcutaneous electri* stimulation[Title/Abstract] OR point embedding [Title/Abstract]))) AND (((poor ovarian response[Title/Abstract] OR poor ovarian responder[Title/Abstract] OR POR[Title/Abstract] OR controlled ovarian hyperstimulation[Title/Abstract] OR reduced ovarian response[Title/Abstract] OR diminished ovarian response[Title/Abstract] OR ovarian stimulation[Title/Abstract] OR diminished ovarian reserve[Title/Abstract])) OR ((low response[Title/Abstract] OR poor responders[Title/Abstract]) AND (in vitro fertilization[Title/Abstract] OR in vitro fertilisation[Title/Abstract])))) AND (((("Randomized Controlled Trials as Topic"[MeSH Major Topic]) OR ("Randomized Controlled Trial"[Publication Type])) OR ((randomized[Title/Abstract] OR radomised[Title/Abstract]))) OR (trial[Title]))

**Result: 34 papers**

**2. Embase**

**Search Date: January 30, 2023**

| No. | Query | Results |
| --- | --- | --- |
| #9 | #6 AND #7 AND #8 | 3 |
| #8 | #1 OR #2 OR #3 | 42678 |
| #7 | 'randomized controlled trial':ti,ab,kw | 138746 |
| #6 | #4 AND #5 | 820 |
| #5 | 'in vitro fertilization':ab,ti OR 'in vitro fertilisation':ab,ti | 36483 |
| #4 | 'poor ovarian response':ab,ti OR 'poor ovarian responder':ab,ti OR 'reduced ovarian response':ab,ti OR 'low response':ab,ti OR 'poor responders':ab,ti | 10472 |
| #3 | acupunct*:ab,ti OR acupress*:ab,ti OR acupoint*:ab,ti OR electroacupunt*:ab,ti OR 'electro acupunt*':ab,ti OR moxdbust*:ab,ti OR 'transcutaneous electri stimulation':ab,ti OR 'point injection':ab,ti OR 'point embedding':ab,ti | 42562 |
| #2 | 'transcutaneous electric nerve stimulation':ti | 138 |
| #1 | 'acupuncture':ti OR 'acupuncture therapy':ti | 25318 |

**Result: 3 papers**

**3. CBM database**

**Search Date: January 30, 2023**

| No. | Query | Results |
| --- | --- | --- |
| #6 | ((((#3) AND (#4))) AND (#5)) | 59 |
| #5 | ((#2) OR (#1)) | 22107 |
| #4 | "随机对照"[常用字段:智能] OR "随机试验"[常用字段:智能] OR "随机"[常用字段:智能] | 2132610 |
| #3 | "针刺"[常用字段:智能] OR "针刺疗法"[常用字段:智能] OR "经皮电刺激"[常用字段:智能] OR "电针"[常用字段:智能] OR "穴位注射"[常用字段:智能] OR "艾灸"[常用字段:智能] OR "穴位埋线"[常用字段:智能] | 224137 |
| #2 | "低反应"[加权:扩展] OR "反应差"[加权:扩展] OR "体外受精"[加权:扩展] | 22107 |
| #1 | "卵巢低反应" [加权:扩展] OR "卵巢反应低下" [加权:扩展] | 0 |

**Result: 59 paper**

**4. CNKI database**

**Search Date: January 30, 2023**

SU=(针刺+针灸+艾灸+电针+经皮电刺激+穴位注射+穴位埋线) AND SU=(卵巢低反应+卵巢反应不良+卵巢反应低下) AND AB=(随机+随机分配+随机对照试验)

**Result: 18 papers**

**5. Wanfang database**

**Search Date: January 30, 2023**

检索表达式(中英文扩展&主题词扩展)：题名或关键词:(针刺 or 针灸 or 艾灸 or 电针 or 穴位注射 or 穴位埋线 or 经皮电刺激) and 题名或关键词:(卵巢低反应 or 卵巢反应低下) and 题名或关键词:(随机 and 对照)

**Result: 1 paper**

**6. VIP database**

**Search Date: January 30, 2023**

(((((((((题名或关键词=针刺 OR 题名或关键词=针灸) OR 题名或关键词=艾灸) OR 题名或关键词=电针) OR 题名或关键词=穴位注射) OR 题名或关键词=穴位埋线) OR 题名或关键词=经皮电刺激) OR 题名或关键词=acupuncture) AND (((题名或关键词=卵巢低反应 OR 题名或关键词=poor ovarian response) OR 题名或关键词=poor responder) OR 题名或关键词=卵巢反应不良)) AND ((((((((题名或关键词=随机对照试验 OR 题名或关键词=randomized clinical trials) OR 题名或关键词=randomized controlled clinical trial) OR 题名或关键词=randomized controlled trial) OR 题名或关键词=randomized controlled trials) OR 题名或关键词=randomized experiment) OR 题名或关键词=rct) OR 题名或关键词=随机对照实验) OR 题名或关键词=随机对照研究))

**Result: 0 paper**

**
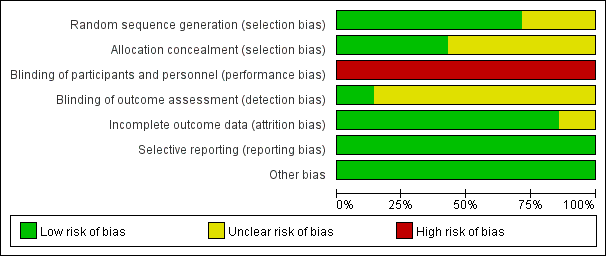
**

**Figure S1.** Review authors' judgments about each risk of bias item presented as percentages across all included studies.

# Forest Plot

**
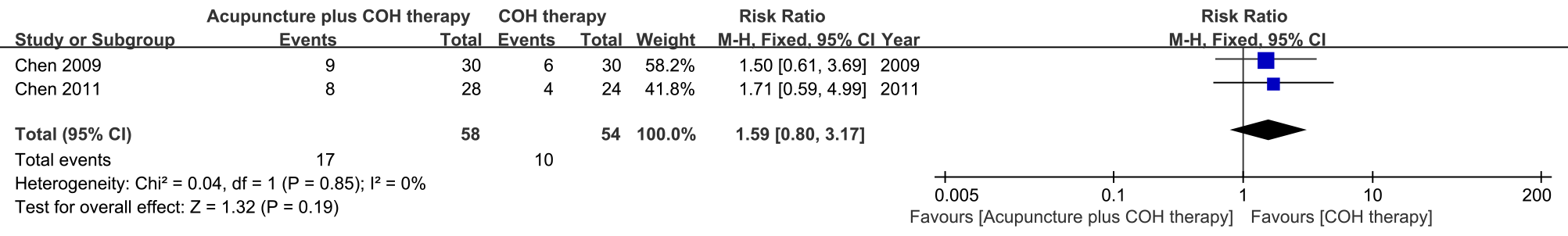
Figure S2.** Forest plot for the clinical pregnant rate of random effect model


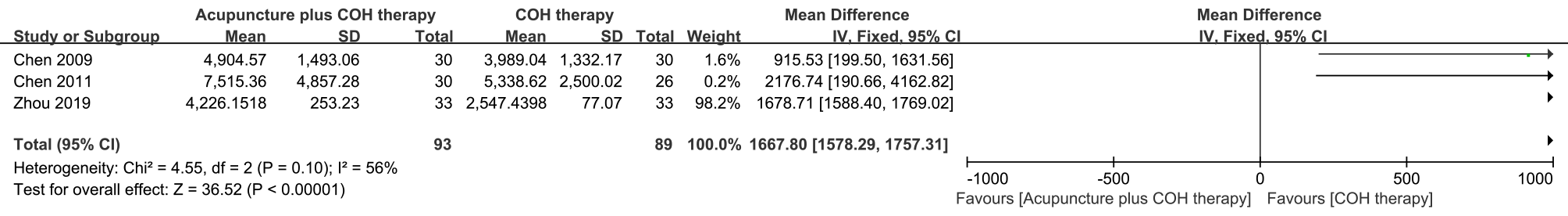
 **Figure S3.** Forest plot for the E_2_ of random effect model

**
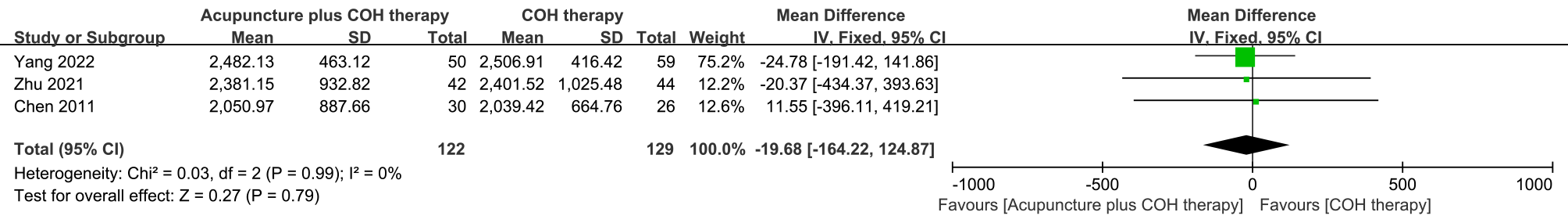
**

**Figure S4.** Forest plot for the dose of Gn of random effect model
